# Extraction-Free Detection of SARS-CoV-2 Viral RNA Using LumiraDx’s RNA Star Complete Assay from Clinical Nasal Swabs Stored in a Novel Collection and Transport Medium

**DOI:** 10.3390/diagnostics13183010

**Published:** 2023-09-21

**Authors:** Luke T. Daum, John D. Rodriguez, Susan R. Ward, James P. Chambers

**Affiliations:** 1 LuJo BioScience Laboratory, 1747 Citadel Plaza, Suite 201, San Antonio, TX 78209, USA; johndr_tn@yahoo.com; 2 LumiraDx, London SW1Y 4LB, UK; susan.ward@lumiradx.com; 3 Department of Biology, University of Texas at San Antonio, San Antonio, TX 78249, USA; jchamber@utsa.edu

**Keywords:** Xtract-Free medium, sample collection, qPCR, diagnostics, COVID-19, SARS-CoV-2, LumiraDx, RNA Star Complete, extraction-free detection, NAT, point of care

## Abstract

**Background:** The rapid detection of severe acute respiratory syndrome coronavirus-2 (SARS-CoV-2) is vital for patient care. The LumiraDx™ SARS-CoV-2 RNA Star Complete (RSC) is an *Emergency Use Authorization*-recognized molecular test using nasal/nasopharyngeal swabs immersed in a viral/universal transport medium (VTM/UTM). However, there is a critical need for an alternative medium for point-of-care testing (POCT). This study aimed to investigate Xtract-Free (XF), a novel collection medium for transport and direct (extraction-free) use with nucleic acid tests. **Methods:** Using serially diluted SARS-CoV-2 viral RNA (vRNA) in a routine UTM and XF, a limit of detection (LOD) was established via an RSC test and a quantitative reverse transcription PCR (RT-qPCR). Additionally, the results obtained from a panel of 108 clinical “car-side” nasal swabs collected in XF during the coronavirus pandemic and assessed using the ”gold-standard” RT-qPCR assay were compared to Lumira’s RSC assay. **Results:** The average replicate RT-qPCR cycle threshold (C_T_) values for vRNA in XF and UTM were observed to be equivalent. An LOD for which five out of five replicates were detected using XF or VTM was approximately 2000 copies/mL. The nasal swabs collected in XF exhibited 93.9% positive percent agreement (sensitivity) and 100% negative percent agreement (specificity) compared to the RT-qPCR. Three specimens tested positive via an RT-qPCR were negative when tested via RSC; however, all three samples had C_T_ values ≥ 36.4. **Conclusions:** XF is equivalent to VTM/UTM and is compatible for use with the RSC test. Furthermore, XF can be used directly with RT-qPCRs and rapid antigen testing without the requirement for separate nucleic acid extraction (an extraction-free process), making it ideal for cost-effective high-throughput and decentralized respiratory testing. **Impact Statement:** This study is the first to evaluate LumiraDx’s SARS-CoV-2 RNA Star Complete assay in concert with Xtract-Free (XF), a novel collection medium containing a proprietary RNase-inactivating technology for the rapid, ”extraction-free” detection of SARS-CoV-2 RNA from clinical nasal swabs. Specimens collected in XF combined with rapid LumiraDx detection provide a safe and sensitive alternative to VTM/UTM, and Molecular Transport medium (MTM) for high throughput, “extraction-free” molecular detection.

## 1. Introduction

Since the severe acute respiratory syndrome coronavirus-2 (SARS-CoV-2) virus was detected in December 2019 in Wuhan, China, the pandemic caused by coronavirus disease 2019 (COVID-19) has quickly become a persistent worldwide endemic [1,2]. Currently, SARS-CoV-2 continues to infect and re-infect individuals via the emergence and spread of antigenically distinct variants [3,4]. To date, there have been more than 690 million cases and 6.8 million deaths attributed to COVID-19 [5]. However, the cumulative global infections are likely underestimated due to asymptomatic cases and unreported self-testing using rapid antigen testing (RAT) [6].

The reverse-transcription quantitative polymerase chain reaction (RT-qPCR) is a nucleic acid test (NAT) that is considerably more sensitive compared to LFAs for detecting SARS-CoV-2 [7,8]. However, an RT-qPCR is labor-intensive and time-consuming (taking several hours before results can be reported) and requires a high-complexity and centralized diagnostic laboratory with technical expertise to perform testing. The Centers for Disease Control and Prevention (CDC) Influenza SARS-CoV-2 Multiplex Assay is one of several RT-qPCR tests that detect SARS-CoV-2 in upper or lower respiratory specimens [9]. Like most lab-developed RT-qPCR tests, there is a requirement for the prefatory nucleic acid extraction of samples collected in a commercial viral or universal transport medium (VTM and UTM). The cobas^®^ Liat^®^ System is a small FDA-approved device for the detection of SARS-CoV-2 and the influenza A and influenza B viruses [10,11,12]. This point-of-care (POC) system is fully automated, including onboard nucleic acid purification and amplification from nasopharyngeal (NP) or VTM and UTM. However, the Liat^®^ can only process one sample at a time and is not suited for high-throughput or centralized respiratory testing. There is a critical need for alternative molecular tests to the RT-qPCR that (1) do not require upfront nucleic acid extraction, (2) offer a rapid turn-around time, and (3) are suited for high-volume, centralized testing, point-of-care testing (POCT), and decentralized mobile formats. A rapid and sensitive alternative to the RT-qPCR would enhance patient care, facilitate the rapid detection of local and regional outbreaks, and aid in detecting emerging variants and re-infections within the community.

The LumiraDx SARS-CoV-2 RNA STAR Complete (RSC; London, UK) is an FDA *Emergency Use Authorization* (EUA)-accepted molecular test for the rapid, qualitative detection of nucleic acid from SARS-CoV-2 [13]. The test is performed using respiratory swabs collected from individuals suspected of harboring SARS-CoV2 and has been widely utilized during the pandemic for direct patient care and contact tracing studies and in self-collect, POC, and mobile health formats. The RCS assay is approved for use on several qPCR platforms including ThermoFisher’s QuantStudio instruments, (Waltham, MA, USA) and is unique compared to most molecular tests since the collected specimens are added directly to the reaction plate after a gentle lysing step. Thus, there is no nucleic acid extraction required prior to testing if the sample is collected in VTM/UTM. Additionally, the RSC thermocycling parameters are rapid, consisting of 30 short cycles of 11 s per cycle. Therefore, using the RSC assay, up to 382 samples can be assessed (plus controls) within 20 min.

Most respiratory collection kits consist of a nasopharyngeal or nasal anterior swab placed into a vial containing a viral or universal transport medium (VTM and UTM). VTMs and UTMs are complex mixtures of sugars, salts, and buffers developed more than two decades ago for viral/bacterial culturing [14,15]. Many VTMs/UTMs contain reagents, i.e., bovine serum albumen and gelatin, that can be carried over and cause inhibition during nucleic acid extraction and nucleic acid amplification (Daum and Chambers, unpublished [16]). Recently, newer molecular transport media (MTMs) such as PrimeStore (Longhorn Vaccines and Diagnostics, Bethesda, MD, USA) and eNat (Copan Diagnostics, Brescia, Italy) were developed to inactivate samples via the chemical lysis of cellular membranes. However, MTMs contain toxic reagents (i.e., guanidine) that are harmful if accidentally ingested [17]. Additionally, these reagents are hazardous to the environment and can release potentially toxic cyanide gas if they come into contact with bleach products during cleanup [18,19].

Additionally, swabs collected in MTM require the extraction of nucleic acids prefatory to detection using automated instrumentation extraction kits containing para-magnetized silica-coated beads or spin columns. For the high-throughput nucleic acid extraction of respiratory samples collected in VTM/UTM and MTM, automated devices such as the QIAsymphony AS (Qiagen, Hilden, Germany) and Roche MagNA Pure 96 System (Roche, Basil, Switzerland) are often utilized. However, high-throughput nucleic acid extraction requires the use of technical manpower and additional costly consumables and reagent kits.

Xtract-Free (XF) is a newly developed biospecimen storage and transport medium for direct, “extraction-free” use. The medium is compatible for direct use with a qPCR, other nucleic acid detection methods, and rapid antigen testing. XF consists of a non-toxic blend of reagents that preserves RNA/DNA and proteins from nuclease degradation upon collection. The reagents in XF are compatible with qPCR “master-mix”; therefore, the collected samples do not require nucleic acid extraction and can be directly added to qPCRs. Alternatively, since XF is guanidine- and alcohol-free, it may also be safely utilized with high-throughput extraction devices that use bleach-based compounds for cleanup between runs.

In this study, the performance of XF was evaluated for use with LumiraDx’s RSC assay. Specifically, this study sought to compare (1) the limit of detection (LOD) using SARS-CoV-2 RNA in XF and UTM and (2) the performance of the RSC assay to results obtained via a “gold standard” RT-qPCR, using a panel of positive and negative clinical specimens collected in XF.

## 2. Methods

### 2.1. Study Population and Clinical Samples

Clinical specimens (*N* = 108) were obtained from nasal flocked swabs (Puritan Medical Devices, Guilford, MA, USA) swirled five times in each nostril and placed into cryotubes containing 1.5 mL of XF (LuJo BioScience Laboratory, San Antonio, TX, USA). All the specimens were originally tested for SARS-CoV-2 shortly after collection using the CDC’s Influenza SARS-CoV-2 Multiplex Assay [9]. This retrospective study using Lumira’s RSC kit was deemed exempt from review by the *Internal Review Board* in accordance with FDA guidance since the testing included informed consent, the results did not impact patient care, and all the samples were de-identified and properly disposed of after use.

### 2.2. RT-qPCR Testing

For the testing comparison, the Influenza SARS-CoV-2 Multiplex Assay was used as described [9]. Briefly, using a QIAamp RNA Viral Mini Kit (Qiagen), a 140 µL specimen was added to 560 µL of Lysis Buffer AVL and subjected to spin-column viral RNA extraction with a 60 μL final elution in Buffer AVE, according to the manufacturer’s recommendations [20]. For the RT-qPCR detection of SARS-CoV-2 RNA, the TaqPath 1-step RT-qPCR MM (ThermoFisher Scientific) was used with the CDC’s primers and probes, targeting N1 and RNaseP on a ThermoFisher QuantStudio 5 instrument. Positive and negative control reactions were included for each RT-qPCR run. During the RT-qPCR analysis, clinical samples that tested positive were recorded according to the cycle threshold (C_T_) value of the N1 viral target. RNaseP, a human gene target, was included as an internal positive control for each specimen tested. Lower C_T_ values indicated a higher initial viral RNA concentration, with a value > 40 indicating *no amplification present*. After the initial qPCR testing, the clinical specimens were stored at −80 °C until later use.

A limit of detection (LOD) was performed using an RT-qPCR and RNA Star Complete via 10-fold serial dilution of purified SARS-CoV-2 viral RNA. For each dilution, a human oral matrix control was included in each medium. The viral copy number was assessed via extrapolation from reference SARS-CoV-2 RNA (American Type Culture Collection, Manassas, VA, USA), using an RT-qPCR standard curve analysis. For the LOD experiments, 5 replicate reactions from each dilution were evaluated. At the lowest dilution at which 5 out of 5 replicates were detected, an additional 20 replicates were performed.

### 2.3. LumiraDx Testing

The LumiraDx SARS-CoV-2 RNA Star Complete assay was performed as described [21]. Briefly, for pre-processing, 24 µL of the specimen was added to a 96-well optical plate containing 4.8 µL of Extraction Buffer, pipetted 10 times, and briefly spun to bring the contents down. To each reaction, 31.2 µL of Reaction Mix (containing 10 µL of Salt Buffer, 1.2 µL of IC/P Mix, and 20 µL of Master Mix) was added to bring the total reaction volume to 60 µL/rxn. The plate was pipetted up/down 10 times and briefly spun before initiating the run. The analysis was performed according to defined thermocycling parameters described previously [21], using a QuantStudio 5 instrument. The instrument’s run time for the RNA Star Complete assay was approximately 20 min [21].

A statistical analysis of the comparator test was performed using MEDCALC23 [22] for the determination of the positive percent agreement (PPA), negative percent agreement (NPA), confidence intervals (CI), accuracy, and disease prevalence.

## 3. Results

### 3.1. Limit of Detection (LOD)

**Figure 1:**
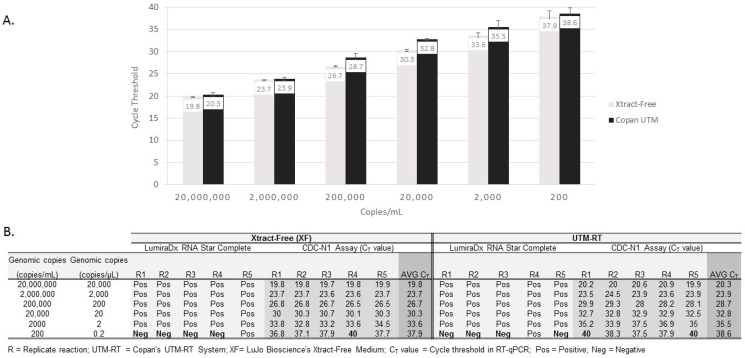
Limits of detection for SARS-CoV-2 virus in Xtract-Free and Copan UTM-RT medium, according to RNA Star Complete and RT-qPCR assays. Panel (**A**): RT-qPCR limit of detection by medium type. An average of five replicates for each dilution with standard deviation bars are shown. Panel (**B**): Limit of detection comparing the RNA Star Complete assay and RT-qPCR in each medium. A cycle threshold value of 40 indicates *No detection*.

Limits of detection (LODs) for the quantified viral SARS-CoV-2 RNA (vRNA) in XF or UMT are shown in Figure 1 (panel A and B). The average C_T_ values for vRNA in a ten-fold reduction series (6-logs) for each medium, obtained using the RT-qPCR, are shown (Figure 1, panel A). According to the RT-qPCR and the RSC assay, five out of five replicates containing SARS-CoV-2 RNA were detected from XF and UTM dilutions containing 20,000,000 copies/mL to 2000 copies/mL (5-logs). At the lowest dilution, i.e., 200 copies/mL (0.2 copies/µL), four out of five replicates (80%) and one out of five (20%) replicates were detected in each medium using the RT-qPCR and LumiraDx’s RSC assay, respectively (Figure 1, panel B). At 2000 copies/mL, the lowest LOD dilution at which five out of five replicates were detected in the XF and UTM (average CDC qPCR C_T_ values of 33.6 and 35.5, respectively), ten additional replicates were detected using the qRT-PCR and the RSC assay and confirmed as positive.

### 3.2. Clinical Evaluation

Of the 108 clinical specimens collected in XF (46 true positives (TPs) and 62 true negatives (TNs) determined via a RT-qPCR), 43 were positive and 65 were negative according to the RSC assay. The positive percent agreement (PPA), defined as the percentage of specimens testing positive among the 46 true positive samples, was 93.9% (C.I. = 83.1–98.7%) compared to the “gold standard” RT-qPCR. The negative percent agreement (NPA), defined as the percentage of specimens testing negative among the 62 true negative samples, was 100% (C.I. = 94.2–100%). There were three false negative (undetected) samples from the RSC assay for which previous positive samples were detected via RT-qPCR when C_T_ values were low, i.e., C_T_ > 34.8, with one sample having a C_T_ value of 39.2. Table 1 summarizes the clinical detection results of the RSC assay according to the PPA, NPA, and accuracy according to the RT-qPCR analysis.

## 4. Discussion

The RSC is an EUA-accepted, non-isothermal nucleic acid test intended for the qualitative detection of SARS-CoV-2 vRNA from respiratory swabs collected from individuals suspected of harboring COVID-19. The test offers several advantages for clinical diagnostic laboratories compared to routine RT-qPCR assays. First, the RST assay is performed directly on original specimens without the need for prefatory nucleic acid extraction. This is critically important because the requirement for nucleic acid extraction is (1) laborious and time-consuming, requiring additional steps for lysis, washing, and separation/removal of cellular debris, and (2) expensive due to the associated costs of extraction kits, pipette tips, and other consumables used during the nucleic acid extraction. Another benefit of the RSC assay is the rapid instrument run time, i.e., the time to result, which is less than 20 min (including reverse transcription) compared to the ~90 min run time of routine RT-qPCRs, including the CDC test used in this study. Furthermore, RSC results are qualitative, i.e., they are shown as a positive or negative result, which provides a simplistic interpretation for laboratory reporting. Lastly, the RSC assay is platform-agnostic and has been validated for use on several qPCR thermocycling instruments.

We show that its detection performance was equivalent for serial dilutions of SARS-CoV-2 vRNA in XF compared to UTM, according to the CDC’s RT-qPCR and Lumira’s RSC test (Figure 1, panel A and B). In a clinical evaluation of nasal swabs in XF, there were three false negative samples using the RSC assay that were initially determined to be positive via the CDC’s RT-qPCR test. However, in these three samples, the qPCR C_T_ values were greater than 34.8, with one sample having a C_T_ value of 39.2. The detection of positive samples using C_T_ values from low-level samples can be problematic since the C_T_ value is dependent on the initial vRNA concentration. The detection of positive samples using C_T_ values from low-level samples can be problematic since the C_T_ value is dependent on the initial vRNA concentration. For example, a high value, e.g., C_T_ = 36, indicates a low concentration of vRNA targets in the sample (Note: a C_T_ value of 45 = no detectable vRNA targets, according to CDC’s RT-qPCR assay). Thus, in an RT-qPCR analysis, a low copy concentration, when a CT value is high, may be reported as “positive” for SARS-CoV-2 despite being negative in other tests such as rapid antigen tests [23,24,25]. The three patients that were missed via the RSC testing but found to be positive via the CDC’s test were from low-target samples in which the C_T_ values were >34.8. Thus, the high C_T_ samples detected via RT-qPCRs are often difficult to detect with other tests [23,24,25].

The primary constraint to the generalization of these results is the limited sample size, i.e., 108 clinical samples used in this study. A larger sample size may provide further insight into the likelihood of additional false positives and false negative samples, which are critical to accurate SARS-CoV-2 detection.

In this study, nasal swabs collected in an Xtract-Free collection medium and tested using LumiraDx’ s RSC assay exhibited equivalent results compared to the results of an RT-qPCR. Importantly, this study supports the use of XF, an alternative collection medium to commercial UTMs/VTMs for use with LumiraDx’s molecular RSC assay. The collection of nasal specimens in XF compliments the LumiraDx assay and offers several important benefits. Sample collection can easily be performed at POC or high-volume settings where a VTM/UTM is limited or unavailable. Furthermore, specimens collected in XF can be used for nucleic acid testing (including an RT-qPCR) with or without extraction. Importantly, the same specimen collected in XF can be further assessed via rapid antigen tests (unpublished, Daum et al., 2023 [16]). In contrast to VTMs/UTMs and MTMs, XF provides a safe collection solution for multi-use testing from a single collection device. This is particularly important within the ongoing COVID-19 pandemic, when diagnostic testing continues to transition from centralized labs to home-use, self-collection, and point-of-care settings.

## Figures and Tables

**Table 1:** Detection of SARS-CoV-2 viral RNA from clinical swabs (*N* = 108) collected in Xtract-Free™ using the LumiraDx RNA Star Complete assay compared with results achieved via a standard RT-qPCR.

LumiraDx RNA Star Complete Kit		
Statistic	Value (%)	95% C.I.
PPA	93.9	83.1 to 98.7%
NPA	100	94.2 to 100%
Accuracy	97.3	92.3 to 99.4%
Disease Prevalence	44.1	34.7 to 53.9%

PPA = positive percent agreement (sensitivity); NPA = negative percent agreement (specificity).

## Data Availability

Not applicable.

## References

[1] Chen N., Zhou M., Dong X., Qu J., Gong F., Han Y., Qiu Y., Wang J., Liu Y., Wei Y. (2020). Epidemiological and clinical characteristics of 99 cases of 2019 novel coronavirus pneumonia in Wuhan, China: A descriptive study. Lancet.

[2] Zhu N., Zhang D., Wang W., Li X., Yang B., Song J., Zhao X., Huang B., Shi W., Lu R. (2020). A Novel Coronavirus from Patients with Pneumonia in China, 2019. N. Engl. J. Med..

[3] Araf Y., Akter F., Tang Y.D., Fatemi R., Parvez M.S.A., Zheng C., Hossain M.G. (2022). Omicron variant of SARS-CoV-2: Genomics, transmissibility, and responses to current COVID-19 vaccines. J. Med. Virol..

[4] Forchette L., Sebastian W., Liu T. (2021). A Comprehensive Review of COVID-19 Virology, Vaccines, Variants, and Therapeutics. Curr. Med. Sci..

[5] COVID-19 Coronavirus Pandemic. Worldometer Website. https://www.worldometers.info/coronavirus/.

[6] Ritchey M.D., Rosenblum H.G., Del Guercio K., Humbard M., Santos S., Hall J., Chaitram J., Salerno R.M. (2022). COVID-19 Self-Test Data: Challenges and Opportunities—United States, October 31, 2021–June 11, 2022. MMWR Morb. Mortal. Wkly. Rep..

[7] Nagura-Ikeda M., Imai K., Tabata S., Miyoshi K., Murahara N., Mizuno T., Horiuchi M., Kato K., Imoto Y., Iwata M. (2020). Clinical Evaluation of Self-Collected Saliva by Quantitative Reverse Transcription-PCR (RT-qPCR), Direct RT-qPCR, Reverse Transcription-Loop-Mediated Isothermal Amplification, and a Rapid Antigen Test To Diagnose COVID-19. J. Clin. Microbiol..

[8] Ferté T., Ramel V., Cazanave C., Lafon M.E., Bébéar C., Malvy D., Georges-Walryck A., Dehail P. (2021). Accuracy of COVID-19 rapid antigenic tests compared to RT-PCR in a student population: The StudyCov study. J. Clin. Virol..

[9] CDC’s Influenza SARS-CoV-2 Multiplex Assay. Updated November 14, 2022. https://www.cdc.gov/coronavirus/2019-ncov/lab/multiplex.html.

[10] Cobas^®^ SARS-CoV-2 & Influenza A/B: Nucleic Acid Test for Use on the Cobas^®^ Liat^®^ System. Available at the Food and Drug Administration Website. https://www.fda.gov/media/142193/download.

[11] Roche Diagnostics (2017). Cobas^®^ Liat^®^ System. Operator’s Manual.

[12] Young S., Phillips J., Griego-Fullbright C., Wagner A., Jim P., Chaudhuri S., Tang S., Sickler J. (2020). Molecular point-of-care testing for influenza A/B and respiratory syncytial virus: Comparison of workflow parameters for the ID Now and cobas Liat systems. J. Clin. Pathol..

[13] LumiraDx Fast Lab Solutions SARS-CoV-2 RNA Star Complete. Instructions for Use. https://www.lumiradx.com/assets/pdfs/fast-lab-solutions/sd-com-art-00071-r.4-lumiradx-sars-cov-2-rna-star-complete-quick-reference-instructions-eua_fdafinal.pdf?v=1.

[14] Copan Diagnostics Copan Universal Transport Medium (UTM). Instructions for Use. https://nvrl.ucd.ie/sites/default/files/uploads/pdfs/UTM-RT_Flocked_Polyester_Swabs.pdf.

[15] Becton Dickinson BD Universal Transport Medium. Instructions for Use. https://www.bd.com/resource.aspx?IDX=14053.

[16] Daum L.T., Chambers J.P. (2023). LuJo BioScience Laboratory LLC., San Antonio, TX.

[17] Ertell K. Pacific Northwest National Laboratory. A Review of Toxicity and Use and Handling Considerations for Guanidine, Guanidine Hydrochloride, and Urea. www.pnnl.gov/main/publications/external/technical_reports/PNNL-15747.pdf.

[18] Centers for Disease Control and Prevention (CDC) Lab Alert: Important Update about Molecular Transport Media (MTM) and Cyanide Gas. www.cdc.gov/locs/2020/important_update_about_mtm_and_cyanide_gas.html.

[19] Lu X., Wang L., Sakthivel S.K., Whitaker B., Murray J., Kamili S., Lynch B., Malapati L., Burke S.A., Harcourt J. (2020). US CDC Real-Time Reverse Transcription PCR Panel for Detection of Severe Acute Respiratory Syndrome Coronavirus 2. Emerg. Infect. Dis..

[20] (2020). Qiagan QIAamp RNA Viral Mini Handbook. www.qiagen.com/dk/resources/resourcedetail?id=c80685c0-4103-49ea-aa72-8989420e3018&lang=en.

[21] (2022). LumiraDx SARS-CoV-2 RNA Star Complete. LumiraDx. https://www.lumiradx.com/assets/pdfs/fast-lab-solutions/sars-cov-2-rna-star-ifu-for-ruo-ous.pdf?v=1.

[22] MEDCALC Statistical Software Diagnostic Test Evaluator Software. www.medcalc.org/calc/diagnostic_test.php.

[23] Pandey A.K., Mohanty A., Hada V., Rath R.S., Kumar S., Kishore S., Kant R. (2021). Comparison of the Rapid Antigen Testing Method With RT-qPCR for the Diagnosis of COVID-19. Cureus.

[24] Wagenhäuser I., Knies K., Rauschenberger V., Eisenmann M., McDonogh M., Petri N., Andres O., Flemming S., Gawlik M., Papsdorf M. (2021). Clinical performance evaluation of SARS-CoV-2 rapid antigen testing in point of care usage in comparison to RT-qPCR. eBioMedicine.

[25] Klajmon A., Olechowska-Jarząb A., Salamon D., Sroka-Oleksiak A., Brzychczy-Włoch M., Gosiewski T. (2021). Comparison of Antigen Tests and qPCR in Rapid Diagnostics of Infections Caused by SARS-CoV-2 Virus. Viruses.

